# Impfhindernisse – Einstellungen von Eltern zur HPV-Impfung

**DOI:** 10.1007/s00103-025-04021-9

**Published:** 2025-03-07

**Authors:** Ariane Kerst, Miriam Gerlich

**Affiliations:** Referat T3 – Sexuelle Gesundheit, Prävention von HIV und anderen STI, Bundesinstitut für Öffentliche Gesundheit (BIÖG), Maarweg 149–161, 50825 Köln, Deutschland

**Keywords:** HPV-Impfung, Impfhindernisse, Impfprävention, Impfmüdigkeit, Impfkommunikation, Impfbereitschaft, Impfakzeptanz, Gesundheitskompetenz, HPV vaccination, Barriers to vaccination, Vaccine hesitancy, Vaccine communication, Vaccine acceptance, Intention to vaccinate, Vaccine confidence, Health literacy

## Abstract

**Hintergrund:**

Obwohl die HPV-Impfung das Risiko von HPV-bedingtem Krebs erheblich senkt, sind die Impfquoten in Deutschland bei Mädchen und Jungen im Alter von 9 bis 14 Jahren immer noch zu niedrig. Zur Steigerung der HPV-Impfquoten ist die Erfassung von Einstellungen von Eltern bzw. Sorgeberechtigten von Kindern im empfohlenen Impfalter wesentlich.

**Methoden:**

Es wurde eine repräsentative Querschnittsbefragung von Eltern bzw. Sorgeberechtigten von Kindern im Alter von 9 bis 15 Jahren durchgeführt (31.05.–19.07.2023). Von besonderem Interesse waren dabei die Einstellungen von jenen Eltern, die ihr Kind (noch) nicht gegen HPV haben impfen lassen (Hauptzielgruppe). Es wurde eine kombinierte Telefon- und Online-Befragung durchgeführt, bei der insgesamt 1439 Elternteile befragt wurden, davon 1000 Elternteile ungeimpfter Kinder.

**Ergebnisse:**

Ein Teil der Eltern ist bzgl. der HPV-Impfung noch unentschlossen oder tendiert dazu, ihr Kind nicht gegen HPV impfen zu lassen (21 % bzw. 8 %). 5 % lehnen die Impfung ab. 23 % der Befragten fühlen sich eher oder sehr schlecht über die HPV-Impfung informiert, 22 % weder gut noch schlecht. Mit Abstand die vertrauenswürdigste Informationsquelle zur HPV-Impfung sind Ärztinnen und Ärzte (85 %). Häufigste genannte Gründe gegen eine HPV-Impfung sind mangelnde öffentliche Aufklärung (59 %), keine empfundene Notwendigkeit für die Impfung zum jetzigen Zeitpunkt (46 %), die Angst vor möglichen Nebenwirkungen (40 %) und die Aussage, dass das Kind später selbst über die Impfung entscheiden solle (39 %).

**Diskussion:**

Die Passgenauigkeit von Kommunikationsmaßnahmen zur HPV-Impfung sollte stetig überprüft und ggf. angepasst werden. Ärztinnen und Ärzte genießen ein hohes Vertrauen und sollten bei der HPV-Impfaufklärung durch Materialien und spezifische Schulungen unterstützt werden.

## Abstract (en)

**Background:**

Although HPV vaccination substantially reduces the risk of HPV-related cancer, vaccination coverage in Germany is still too low among girls and boys aged 9 to 14 years. To increase HPV vaccination coverage, it is essential to assess the attitudes of parents or guardians of children of recommended vaccination age.

**Methods:**

A representative cross-sectional survey of parents or guardians of children aged 9 to 15 years was conducted (31 May–19 July 2023). Of particular interest were the attitudes of those parents who had not (yet) had their child vaccinated against HPV (primary target group). A combined telephone and online survey was conducted in which a total of 1439 parents were interviewed, including 1000 parents of unvaccinated children.

**Results:**

A portion of the parents is still undecided about the HPV vaccination or tend to be reluctant to have their child vaccinated against HPV (21% and 8%, respectively). 5% are opposed to the vaccination, 23% feel rather or very poorly informed about the HPV vaccination, and 22% feel neither well nor poorly informed. By far the most trusted source of information on HPV vaccination are doctors (85%). The most frequently cited reasons against HPV vaccination are a lack of public education (59%), no perceived need for vaccination at this time (46%), fear of possible side effects (40%), and the statement that the child should decide for themselves about vaccination later (39%).

**Discussion:**

The suitability of communication measures for HPV vaccination should be continuously reviewed and adjusted if necessary. Doctors are highly trusted and should be supported in providing information on HPV vaccination through materials and specific training.

## Einleitung

Humane Papillomviren (HPV) gehören zu den verbreitetsten sexuell übertragbaren Erregern und umfassen über 200 verschiedene Virustypen, bei denen man zwischen Niedrigrisiko- und Hochrisiko-Typen unterscheidet. Niedrigrisiko-Typen können Warzen im Anogenitalbereich oder seltener auch im Mund-Rachen-Raum verursachen. Hochrisiko-Typen sind hingegen maßgeblich an der Entstehung von Karzinomen und deren Vorstufen am Gebärmutterhals (Zervix) beteiligt. Die meisten HPV-Infektionen sind nach einer Zeitspanne von bis zu 18 Monaten nicht mehr nachweisbar, sie können aber auch über Jahre persistieren [1]. Etwa 10 % der Infektionen mit Hochrisiko-Typen bleiben bestehen und können viele Jahre bis Jahrzehnte später zu Krebsvorstufen oder einem Karzinom führen. HPV-bedingter Krebs kann auch an weiteren Geschlechtsorganen (Vulva, Vagina oder Penis), am Anus oder im Mund-Rachen-Raum auftreten [2]. Zervixkarzinome werden dabei zu fast 100 % durch HPV verursacht [1]. Gebärmutterhalskrebs gehört zu den häufigsten Krebserkrankungen bei Frauen weltweit. In Deutschland erkrankten im Jahr 2019 etwa 4575 Frauen an einem Zervixkarzinom [3]. Dabei ist Gebärmutterhalskrebs größtenteils vermeidbar und auch heilbar, wenn er früh erkannt und behandelt wird.

Die HPV-Impfung senkt das Risiko von HPV-bedingtem Krebs erheblich. Seit 2007 wird die Impfung für Mädchen und seit 2018 auch für Jungen zwischen 9 und 14 Jahren von der Ständigen Impfkommission (STIKO) empfohlen. Wenn die Impfung bis dahin nicht erfolgt ist, empfiehlt die STIKO eine Nachimpfung bis zum 18. Geburtstag. Die Krankenkassen übernehmen die Kosten im Rahmen der Empfehlung. Einige Krankenkassen erstatten die Impfung auch noch nach dem 18. Geburtstag. Die hohe Effektivität der Impfung wurde in Studien belegt [4]. Die Impfquoten sind jedoch in Deutschland und auch in einigen anderen europäischen Ländern immer noch zu niedrig. Mögliche Barrieren für die HPV-Impfung sind vielfältig und betreffen u. a. Einstellungen, Wissen und Vertrauen von Eltern.

Im Jahr 2023 waren 55 % der 15-jährigen Mädchen und 34 % der 15-jährigen Jungen in Deutschland vollständig gegen HPV geimpft. Mehr als 2 Drittel der 15-jährigen Mädchen (68 %) hatten eine Impfserie zumindest begonnen [5]. In anderen europäischen Ländern variieren die Impfquoten zwischen ca. 5 % und 95 % [6]. Im europäischen Vergleich schneidet Deutschland eher schlecht ab [7].

Die Weltgesundheitsorganisation (WHO) hat im Jahr 2020 eine globale Strategie zur Eliminierung von Gebärmutterhalskrebs verabschiedet [8]. Das Ziel ist eine Inzidenz von unter 4 pro 100.000 Frauen. Dieses soll durch 3 tragende Maßnahmen in allen Ländern bis zum Jahr 2030 erreicht werden:

- eine HPV-Impfquote von 90 % der Mädchen bis zum Alter von 15 Jahren,
- das HPV-Screening bei rund 70 % der Frauen im Alter von 35 Jahren und
- die Behandlung von 90 % der Frauen mit Formen von HPV-bedingtem Krebs oder dessen Vorstufen.

Das europäische Verbundprojekt „PartnERship to Contrast HPV“ (PERCH) knüpft an die globale Strategie der WHO an. Am Projekt beteiligen sich 18 europäische Länder mit 34 Partnerorganisationen [9].

Die Bundeszentrale für gesundheitliche Aufklärung (BZgA), jetzt Bundesinstitut für Öffentliche Gesundheit (BIÖG), leitet eines der 7 Arbeitspakete des Projekts (Arbeitspaket 7 „Training and support in vaccine communication“) und ist an weiteren Arbeitspaketen beteiligt. Die Durchführung erfolgt in enger Zusammenarbeit mit dem Robert Koch-Institut (RKI) und weiteren beteiligten Institutionen.

Das Projekt beabsichtigt eine Steigerung von Wissen und Aufmerksamkeit für HPV-bedingten Krebs und möchte so zu erhöhter Impfakzeptanz beitragen. Das Projekt soll informierte Impfentscheidungen unterstützen und Zugänge zur HPV-Impfung erleichtern. Somit zielt es auch auf die Gesundheitskompetenz von relevanten Zielgruppen, welche deren Motivation, Wissen und Fähigkeit umfasst, relevante Gesundheitsinformationen zu finden, zu verstehen, zu beurteilen und im Alltag anzuwenden [10]. Aufgrund des empfohlenen Impfalters von 9–14 Jahren werden zur HPV-Impfung insbesondere Eltern bzw. Sorgeberechtigte von Kindern und Jugendlichen adressiert.

Um zielgruppenspezifisch zur HPV-Impfung kommunizieren zu können und geeignete Maßnahmen zur Erhöhung der Impfakzeptanz zu entwickeln, ist das Wissen über die Einstellungen, Vorbehalte sowie Bedenken bei Eltern bzw. Sorgeberechtigten von Kindern und Jugendlichen im empfohlenen Impfalter essenziell. Von besonderem Interesse sind dabei die Einstellungen von jenen Eltern bzw. Sorgeberechtigten, die ihr Kind (noch) nicht gegen HPV haben impfen lassen. Um die Konzeption einer zielgerichteten Ansprache zu ermöglichen, wurden diese Daten im Rahmen von PERCH (Arbeitspaket 6 „Improving knowledge and awareness to increase vaccine uptake in target communities“) erhoben und ausgewertet. Teile der Ergebnisse werden hier präsentiert.

## Methode

Im Rahmen des Projekts PERCH wurde eine repräsentative Querschnittsbefragung von Eltern bzw. Sorgeberechtigten von Kindern im für die HPV-Impfung empfohlenen Alter durchgeführt. Die Befragung fand vom 31.05. bis 19.07.2023 statt. Die Grundgesamtheit bildete dabei die deutschsprachige Wohnbevölkerung, in deren Haushalt Kinder im Alter zwischen 9 und 15 Jahren leben. Im Fokus der Befragung standen jene Eltern, die ihr Kind (noch) nicht gegen HPV haben impfen lassen. Diese Zielgruppe wurde über ein Eingangsscreening in der Erhebung ermittelt. Die Merkmale der Stichprobe sind in den Tab. 1 und 2 dargestellt.

**Tab. 1:** Geschlecht, Alter und formale Bildung von Eltern geimpfter und ungeimpfter Kinder

	Elternteile geimpfter Kinder [%]	Elternteile ungeimpfter Kinder [%]
*Anzahl befragter Elternteile*	*N* = 439	*N* = 1000
*Geschlecht des Elternteils*		
Weiblich	61	49
Männlich	39	51
*Alter des Elternteils*		
Unter 30 Jahren	0	1
30–44 Jahre	45	56
45–59 Jahre	54	41
60+ Jahre	1	2
*Formale Bildung des Elternteils*		
(Fach‑)Abitur, Studium	48	40
Mittlerer Schulabschluss	37	37
Hauptschulabschluss	14	21
Kein formaler Schulabschluss	0	1
*Deutsch als Muttersprache*		
Ja	92	91
Nein	7	9

Anmerkung: Aus Gründen der geringen Nennung von „divers“ wurden bei der Auswertung nur die Gruppen Frauen und Männer dargestellt (in der Befragung gestellte Frage: Welchem Geschlecht ordnen Sie sich zu?). Für die Auswertung wurden die Altersgruppen in 2 Gruppen zusammengefasst (bis 44 Jahre; 45 Jahre und älter). Formale Bildung wurde für die Auswertung ebenfalls in 2 Gruppen unterteilt („kein (Fach‑)Abitur“ und „(Fach‑)Abitur“)

**Tab. 2:** Alter, Geschlecht und Anzahl im Haushalt lebender Kinder der Gesamtstichprobe

Alter des Kindes	9–12 Jahre: 57 %
	13–15 Jahre: 43 %
Geschlecht des Kindes	Weiblich: 49 %
	Männlich: 51 %
Anzahl der Kinder im Haushalt (Alter: 9–15 Jahre)	1 Kind: 61 %
	2 Kinder: 35 %
	3 oder mehr Kinder: 5 %

Für die Befragung wurde ein Mixed-Mode-Design ausgewählt, welches computergestützte telefonische Interviews (CATI) sowie computergestützte webbasierte Online-Interviews (CAWI) beinhaltete. Die Befragung wurde vom Markt- und Meinungsforschungsinstitut INFO GmbH im Auftrag der BZgA, jetzt BIÖG, durchgeführt.

### Erhebungsinstrument

Die Konzeption des verwendeten Fragebogens erfolgte im Rahmen des PERCH-Projekts in enger Abstimmung der Projektpartner. Es existieren unterschiedliche Skalen zur Erfassung der Impfbereitschaft (z. B. die „7-Component Vaccination Readiness Scale“, der Fragebogen zu „Parent Attitudes about Childhood Vaccines“ (PACV) und andere [11–16]). Um die Bedarfe und Fragestellungen des Projekts zielgerichtet abzudecken, wurde jedoch ein eigener Fragebogen unter der Federführung der Arbeitspaketleitung erstellt. Da Befragungen in verschiedenen europäischen Partnerländern erfolgt sind, wurde ein englischsprachiger Fragebogen für alle Projektpartner zur Verfügung gestellt. Dieser wurde für den Einsatz in Deutschland inhaltlich adaptiert und übersetzt, was so im Prozess vorgesehen war. Das Erhebungsinstrument wurde dann in Abstimmung mit dem Markt- und Meinungsforschungsinstitut finalisiert. Es bestand aus jeweils einem abgestimmten Einführungstext für die Online- bzw. Telefonbefragung sowie 26 Fragen mit folgenden inhaltlichen Schwerpunkten:

- Impfabsicht und Impfbereitschaft,
- Wissensstand zu HPV bzw. zur HPV-Impfung,
- genutzte Informationsquellen und deren eingestufte Vertrauenswürdigkeit,
- eingestufte Wichtigkeit von Informationen zu HPV bzw. zur HPV-Impfung,
- Gründe gegen eine HPV-Impfung,
- Zugang zur HPV-Impfung (z. B. Erreichbarkeit einer ärztlichen Praxis).

Es wurden geschlossene Fragen genutzt, die entweder die Auswahl einer oder mehrerer Antwortoptionen oder die Einstufung von Items auf einer 4‑ oder 5‑stufigen Skala beinhalteten. Es wurde stets eine Ausweichkategorie (z. B. weiß nicht/keine Antwort) angeboten. Einzelne Fragen beinhalteten zusätzlich die Möglichkeit für Freitext bzw. einen Kommentar.

Zusätzlich wurden folgende soziodemografische Merkmale erfasst: Altersgruppe, Geschlecht, Anzahl und Alter der Kinder (im Rahmen der Spanne des Zielalters), formale Bildung, Muttersprache Deutsch (Ja, Nein; als einfacher Indikator für Migrationshintergrund; Tab. 1 und 2).

Vor der Erhebung im Hauptfeld wurde ein Pretest als computergestützte telefonische Befragung realisiert. Insgesamt wurden 13 Interviews geführt mit dem Ziel zu überprüfen, ob die Fragen verständlich, eindeutig und flüssig gestellt waren. Der Pretest bestätigte die Eignung des Fragebogens für die Erhebung. Im Vorfeld der Haupterhebung wurden lediglich geringfügige Anpassungen in den Formulierungen vorgenommen.

### Rekrutierung der Stichprobe

Grundgesamtheit für die repräsentative Elternbefragung war zunächst die deutschsprachige Wohnbevölkerung, in deren Haushalt Kinder bzw. Jugendliche im Alter zwischen 9 und 15 Jahren leben. Insgesamt wurden 1439 Elternteile im Hauptfeld befragt, davon 1000 Elternteile ungeimpfter Kinder. Wenn mehrere Kinder aus der Zielgruppe in einem Haushalt lebten, wurde das betreffende Kind zufällig mittels eines programmierten Auswahlbefehls im Fragebogen ausgewählt.

Im Zuge eines Eingangsscreenings wurden allen Eltern von Kindern zwischen 9 und 15 Jahren 3 Fragen zur HPV-Impfung gestellt, unabhängig davon, ob das ausgewählte Kind bereits gegen HPV geimpft war oder nicht. Diese 3 Fragen bezogen sich auf den Impfstatus bzw. die Impfabsicht, die gefühlte Informiertheit und die Wichtigkeit von Informationen. Bei allen Eltern wurden sozidemografische Merkmale erfasst.

Nach dem Eingangsscreening erfolgte der Drop-out von Eltern, die ihr Kind bereits gegen HPV hatten impfen lassen („Mein Kind ist bereits gegen HPV geimpft“). Den ausführlichen Fragebogen mit 26 Fragen haben nur Eltern bzw. Sorgeberechtigte erhalten, deren Kind (noch) nicht gegen HPV geimpft war. In dieser Hauptzielgruppe wurden 1000 Elternteile befragt.

Die Interviews wurden zu gleichen Teilen (jeweils *n* = 500) computergestützt telefonisch (CATI) bzw. online (CAWI) durchgeführt, um mögliche Verzerrungen von alleinigen Telefon- oder Online-Befragungen zu reduzieren.

Die Ziehung der Telefonstichprobe erfolgte mit zufallsgenerierten Telefonnummern. Insgesamt wurden 815 Interviews realisiert, davon 500 mit Eltern ungeimpfter Kinder (Nettostichprobe). Unter Berücksichtigung des Screeningeffekts entsprach dies einer Ausschöpfung von ca. 15 % innerhalb der Zielgruppe der Eltern.

Die Teilnehmenden für die Online-Elternbefragung wurden aus dem aktiv rekrutierten Online-Access-Panel gewonnen. Die Zielgruppe wurde zunächst vorselektiert und zudem durch ein vorgeschaltetes Screeninginterview identifiziert. Anonymität wurde bei den Befragungen sichergestellt. Es wurden insgesamt 624 Elternteile online befragt, davon 500 Elternteile ungeimpfter Kinder. Für die CAWI-Stichprobe lässt sich eine Response-Rate von ca. 36 % innerhalb der vorselektierten Zielgruppe schätzen.

Die Auswahl der Probanden erfolgte proportional zur Grundgesamtheit in beiden Teilstichproben (CATI und CAWI). Auftretende strukturelle Disproportionalitäten wurden durch eine Gewichtung der Gesamtstichprobe an die Normstrukturen angepasst.

### Auswertung

Die Daten wurden in anonymisierter Form verarbeitet. Folgende Untersuchungsgruppen wurden ausgewertet: Alter des ausgewählten Kindes (9–12 Jahre, 13–15 Jahre), Geschlecht des ausgewählten Kindes (weiblich, männlich), Alter des Elternteils (bis 44 Jahre, 44 Jahre und älter), Geschlecht des Elternteils (weiblich, männlich), Deutsch als Muttersprache (ja, nein), formale Bildung (kein (Fach‑)Abitur, (Fach‑)Abitur), HPV-Impfung beabsichtigt (auf jeden Fall/eher ja, unentschlossen, eher nicht/auf keinen Fall, bereits geimpft), geschätztes Risiko einer HPV-Ansteckung (sehr/eher hoch, weder hoch, noch gering, eher/sehr gering). Die hier gezeigte Einteilung der Untersuchungsgruppen erfolgte datenbasiert im Anschluss an die Erhebung mit dem Zweck, auswertbare Gruppengrößen zu identifizieren und darzustellen.

Die Auswertungen erfolgten auf Basis bivariater Statistik. Die jeweiligen Anteile wurden einem 2‑seitigen Signifikanztest (T-Test) unterzogen. Dazu wurden paarweise Vergleiche von Spaltenanteilen – also zwischen verschiedenen Auswertungs- bzw. Kopfgruppen – berechnet, um zu eruieren, welche Spaltenpaare (für eine bestimmte Zeile) sich signifikant unterscheiden. Signifikante Unterschiede wurden auf dem 0,05-Signifikanzniveau berechnet. Um das beobachtete Signifikanzniveau für Mehrfachvergleiche anpassen zu können, wurde die Bonferroni-Korrektur genutzt.

Um mögliche Verzerrungen auszugleichen, wurde die Stichprobe nach den Merkmalen Alter, Geschlecht, höchster Schulabschluss und Bundesland an die aus der amtlichen Statistik bekannten Sollstrukturen angepasst. Die statistische Auswertung erfolgte durch die INFO GmbH im Auftrag der BZgA, jetzt BIÖG, mit der Statistiksoftware IBM SPSS Statistics 24 (IBM, Armonk, NY, USA).

## Ergebnisse

### Impfstatus und Impfabsicht

Elternteile der Gesamtstichprobe (*n* = 1439) wurden zum Impfstatus bzw. zur Impfabsicht bzgl. der HPV-Impfung befragt. Die HPV-Impfquote der in die Befragung einbezogenen Kinder liegt bei 29 %. Bei den 13- bis 15-Jährigen (44 %) ist sie signifikant höher als bei den 9‑ bis 12-Jährigen (18 %). Häufiger geimpft sind auch Mädchen (33 %) im Vergleich zu Jungen (26 %). Kinder von Eltern mit höherer Formalbildung sind signifikant häufiger geimpft als jene von Eltern mit niedrigerer Formalbildung (33 % vs. 26 %). Kinder von Eltern, deren Muttersprache nicht Deutsch ist, sind mit 15 % signifikant weniger häufig geimpft (Muttersprache Deutsch: 31 %).

**Abb. 1:**
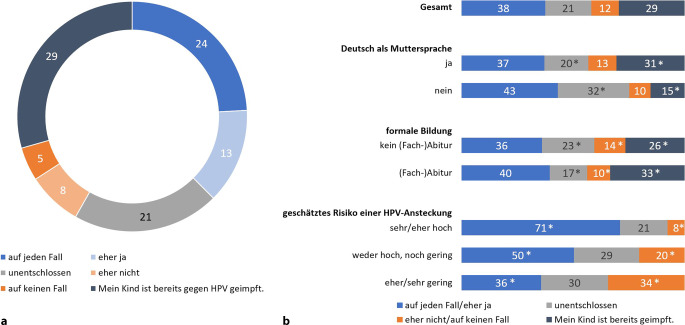
Impfstatus und Absicht zur HPV-Impfung für die Gesamtstichprobe (**a**) und verschiedene Auswertungsgruppen (**b**). Angaben in %. Basis *n* = 1439 befragte Elternteile (alle Befragten). In der Befragung gestellte Frage: „Beabsichtigen Sie, Ihr Kind in Zukunft gegen HPV impfen zu lassen?“ Antworten auf einer 5‑stufigen Skala von „auf jeden Fall“ bis „auf keinen Fall“ mit zusätzlicher Antwortmöglichkeit „Mein Kind ist bereits gegen HPV geimpft“. Signifikante Unterschiede sind mit *Asterisk* gekennzeichnet. Quelle: eigene Abbildung

Die Mehrheit der befragten Eltern steht dem Impfen allgemein (eher) befürwortend gegenüber (77 %). Ablehnend waren 7 % der Befragten. Abb. 1 zeigt den Impfstatus und die Impfabsicht der befragten Eltern zur HPV-Impfung.

Ein signifikanter Unterschied zeigt sich hier zwischen den Geschlechtern. Eltern eines Sohns sind häufiger unentschlossen als Eltern von Mädchen (24 % vs. 17 %, ohne Abbildung). Auch bei niedriger Formalbildung ist die Unentschlossenheit bzgl. der HPV-Impfung signifikant höher als bei höherer Formalbildung (23 % vs. 17 %). Häufiger unentschlossen sind außerdem Eltern, deren Muttersprache nicht Deutsch ist. Fast ein Drittel dieser Befragten (32 %) ist unentschlossen, ob das eigene Kind noch gegen HPV geimpft werden soll (Muttersprache Deutsch: 20 %).

Bei Eltern ungeimpfter Kinder, die das Risiko einer HPV-Infektion als (eher) hoch einschätzen, ist die Impfabsicht fast doppelt so hoch wie bei denjenigen, die von einem (eher) geringen Risiko ausgehen (71 % vs. 36 %). Eltern, die das Risiko einer HPV-Impfung als (eher) gering einschätzen, zeigen die signifikant geringste Impfbereitschaft (36 %) und äußern sich am häufigsten ablehnend (34 %).

### Gefühlte Informiertheit

**Abb. 2:**
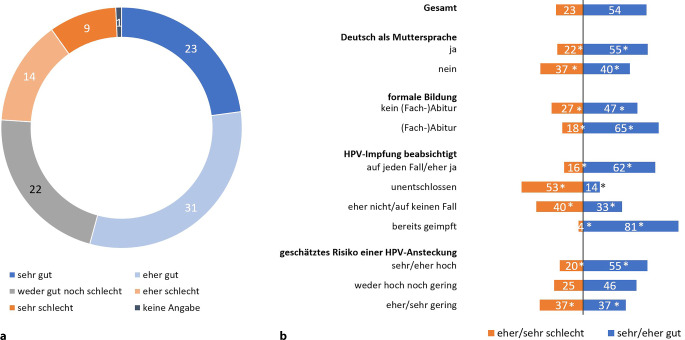
Gefühlte Informiertheit zur HPV-Infektion/-Impfung für die Gesamtstichprobe (**a**) und verschiedene Auswertungsgruppen (**b**). In der Befragung gestellte Frage: „Wie gut fühlen Sie sich zur HPV-Infektion und zur HPV-Impfung informiert?“ Antworten auf einer 5‑stufigen Skala von „sehr gut“ bis „sehr schlecht“. Angaben in %. Basis *n* = 1439 befragte Elternteile (alle Befragten). Signifikante Unterschiede sind mit *Asterisk* gekennzeichnet. Quelle: eigene Abbildung

Abb. 2 zeigt die gefühlte Informiertheit der befragten Eltern. Diese Frage wurde sowohl Eltern geimpfter als auch ungeimpfter Kinder gestellt. 54 % fühlen sich sehr gut oder eher gut über die HPV-Impfung informiert. Signifikante Gruppenunterschiede zeigen sich hier bezüglich der Formalbildung: Während sich 65 % der befragten Eltern mit (Fach‑)Abitur sehr oder eher gut informiert fühlen, sagen dies nur 47 % derjenigen ohne (Fach)-Abitur.

Darüber hinaus zeigen sich Gruppenunterschiede bezogen auf die Muttersprache. Personen, deren Muttersprache nicht Deutsch ist, fühlen sich signifikant häufiger sehr oder eher schlecht informiert im Vergleich zu Befragten, deren Muttersprache Deutsch ist (37 % vs. 22 %).

Auch bei der Impfabsicht zeigen sich Gruppenunterschiede. Insbesondere die unentschlossenen Eltern, aber auch Eltern, die ihr Kind eher nicht oder auf keinen Fall gegen HPV impfen lassen möchten, fühlen sich eher oder sehr schlecht informiert (53 % bzw. 40 %).

### Informationsbeschaffung und Vertrauen in Informationsquellen

Die häufigste Informationsquelle zur HPV-Impfung für Eltern ungeimpfter Kinder sind Kinder- und Jugendärztinnen und -ärzte (42 %). Deutlich seltener werden Gynäkologinnen und Gynäkologen (17 %) sowie Hausärztinnen und Hausärzte hierzu konsultiert (17 %).

Suchmaschinen im Internet, wie z. B. Google, werden von 20 % der Befragten genutzt. Über Websites von offiziellen Gesundheitseinrichtungen informieren sich 16 % der Eltern. Noch nie von der HPV-Impfung gehört haben 11 % der Eltern und 9 % kennen sie nur dem Namen nach. Bei den unentschlossenen Eltern sind es 22 %, die angeben, noch nie von der HPV-Impfung gehört zu haben.

Befragte mit höherer Formalbildung nennen insgesamt mehr Informationsquellen als Befragte mit formal geringerer Bildung, die zudem signifikant häufiger angeben, HPV nur dem Namen nach zu kennen (11 % vs. 6 %).

**Abb. 3:**
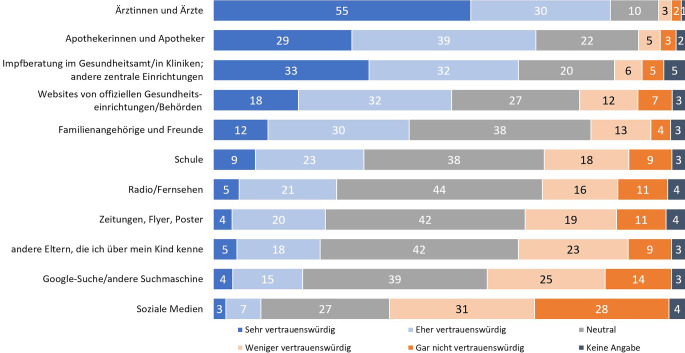
Vertrauen in Informationsquellen. In der Befragung gestellte Frage: „Wie vertrauenswürdig schätzen Sie folgende Informationsquellen ein, um ausreichende Informationen über die HPV-Impfung zu erhalten?“ Antworten auf einer 5‑stufigen Skala von „sehr vertrauenswürdig“ bis „gar nicht vertrauenswürdig“. Angaben in %. Basis *n* = 1000 Elternteile ungeimpfter Kinder. Quelle: eigene Abbildung. Anmerkung: Bei der Auflistung der Informationsquellen ist anzumerken, dass diese ganz verschiedene Absenderschaften haben können (z. B. Fernsehen, Zeitungen oder Flyer). Aus Gründen der Befragungsdauer konnte keine detaillierte Auflistung möglicher Absenderschaften aufgeführt werden. Bei Webseiten wurde unterschieden zwischen öffentlichen Gesundheitseinrichtungen/Behörden und anderen Webseiten

Auch das höchste Vertrauen in Informationsquellen zu HPV und zur HPV-Impfung bekommen Ärztinnen und Ärzte (85 %), gefolgt von Apothekerinnen und Apothekern (68 %) und Impfberatungsstellen im Gesundheitsamt/Kliniken/anderen zentralen Einrichtungen (65 %; Abb. 3).

Die Informationen auf offiziellen Webseiten öffentlicher Gesundheitseinrichtungen oder Behörden halten 18 % für sehr vertrauenswürdig und 32 % für eher vertrauenswürdig. Ein recht hoher Anteil der Befragten vertraut ihnen nicht (19 %). Dem Internet im Allgemeinen und vor allem den sozialen Medien misstraut die große Mehrheit. Von den Befragten halten nur 10 % diese Quellen für eher oder sehr vertrauenswürdig (Abb. 3).

### Gründe gegen eine HPV-Impfung

**Abb. 4:**
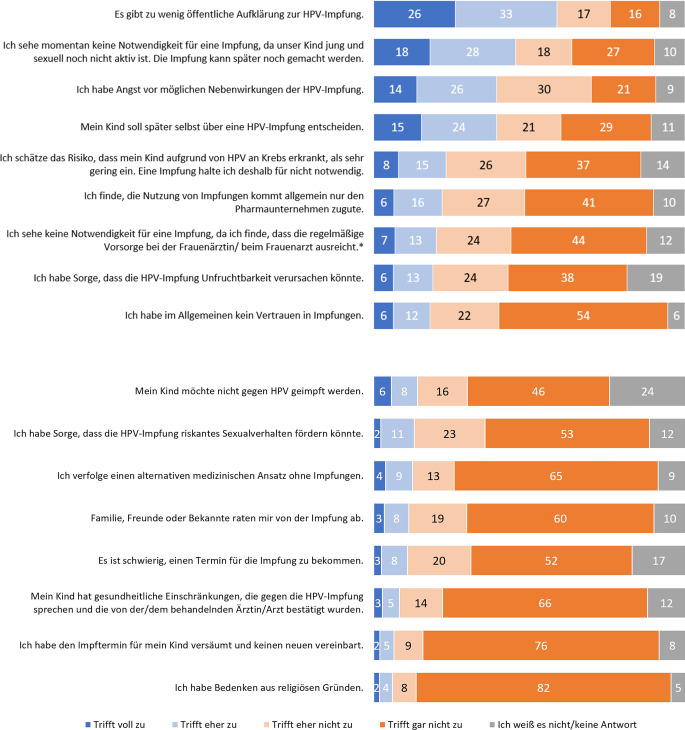
Zustimmung zu möglichen Gründen gegen eine HPV-Impfung. In der Befragung gestellte Frage: „Die folgenden Aussagen können Gründe gegen eine HPV-Impfung sein. Bitte geben Sie für jede Aussage an, inwieweit diese für Sie zutrifft.“ Antworten auf einer 4‑stufigen Skala von „trifft voll zu“ bis „trifft gar nicht zu“. Angaben in %. Basis *n* = 1000 Elternteile ungeimpfter Kinder. *Asterisk* Basis: Elternteile weiblicher ungeimpfter Kinder (*n* = 428). Quelle: eigene Abbildung

Eltern ungeimpfter Kinder wurden gebeten, 17 Aussagen einzuordnen, die gegen eine HPV-Impfung sprechen können (Abb. 4).

Die Aussage: „Es gibt zu wenig Aufklärung zur HPV-Impfung“, trifft insgesamt mit 59 % auf die größte Zustimmung unter den Befragten („trifft voll zu“: 26 %, „trifft eher zu“ 33 %). Gefolgt wird diese von der Aussage: „Ich sehe momentan keine Notwendigkeit für eine Impfung, da mein Kind jung und sexuell noch nicht aktiv ist“ (46 %). Jeweils 40 % bzw. 39 % stimmen den Aussagen: „Ich habe Angst vor möglichen Nebenwirkungen der HPV-Impfung“ und „Mein Kind soll später selbst über eine HPV-Impfung entscheiden“, voll oder eher zu.

Weiteren möglichen Gründen gegen eine HPV-Impfung stimmen weniger als ein Viertel der Befragten voll oder eher zu. Die geringste Zustimmung erhalten die Aussagen: „Mein Kind hat gesundheitliche Einschränkungen, die gegen eine HPV-Impfung sprechen“ (8 %), „Ich habe den Impftermin für mein Kind versäumt und keinen neuen vereinbart“ (7 %) und „Ich habe Bedenken aus religiösen Gründen“ (6 %).

Ein grundsätzliches Misstrauen in Impfungen äußerten 18 % der Eltern. Ein Teil der Befragten (13 %) verfolgt einen „alternativen medizinischen Ansatz ohne Impfungen“. Zudem stimmen 22 % der Eltern zu, dass Impfungen hauptsächlich den Pharmaunternehmen zugutekämen.

## Diskussion

In Deutschland sind immer noch zu wenige Kinder und Jugendliche im empfohlenen Alter gegen HPV geimpft. Die vorliegenden Ergebnisse zeigen, dass ein Teil der Eltern zur HPV-Impfung noch unentschlossen ist oder dazu tendiert, ihr Kind eher nicht gegen HPV impfen zu lassen (21 % bzw. 8 %). Eine frühere Befragung zu Einstellungen von Eltern gegenüber Impfungen allgemein zeigte eine ähnliche Zahl von unentschlossenen Eltern (17 %; [17]). Insgesamt befürwortet jedoch die Mehrheit der Eltern die Durchführung von Schutzimpfungen, wobei die HPV-Impfung von Eltern als weniger wichtig angesehen wird als andere Schutzimpfungen im Kindes- und Jugendalter [17]. Global gesehen ist die Akzeptanz von Impfungen eher die Norm. Nur ein kleiner Teil der Gesamtbevölkerung spricht sich gegen Impfungen aus [18]. Im Jahr 2022 standen 3 % der Befragten in Deutschland Impfungen allgemein (eher) ablehnend gegenüber [17]. In der hier durchgeführten Befragung waren es 5 %, die ihr Kind auf keinen Fall gegen HPV-Impfung impfen lassen möchten. Die Zahl der Ablehnenden ähnelt sich in diesen Befragungen. Teilweise sind Eltern auch nur gegen manche der empfohlenen Impfungen oder es herrscht Unsicherheit und Zögern, was sich auf einzelne Aspekte einer Impfung beziehen kann, z. B. auf den empfohlenen Zeitpunkt der Impfung [18]. Der Zeitpunkt im Impfkalender und der Übertragungsweg spielen auch bei der HPV-Impfung eine Rolle. 46 % der Befragten gaben an, dass die Impfung noch später durchgeführt werden könne, da das Kind noch nicht sexuell aktiv sei. Dies kann dazu führen, dass die Impfung vergessen oder erst nach den ersten sexuellen Kontakten durchgeführt wird. Gerade im Übergang von der Kindheit zur Jugend werden pädiatrische Praxen nicht mehr so regelhaft aufgesucht. Vor diesem Hintergrund ist es besonders wichtig, gesetzlich verankerte Früherkennungsuntersuchungen, wie die Jugenduntersuchung J1, zu nutzen, um den Impfstatus zu überprüfen und die HPV-Impfung ggf. aktiv anzusprechen. Auch die Anerkennung der Kinderuntersuchung U11 bzw. einer zusätzlichen Früherkennungsuntersuchung für 9‑ bis 10-Jährige als Regelleistung erscheint als Möglichkeit zur Steigerung der HPV-Impfquoten. Eine aktuelle Bestandsaufnahme des RKI hat zudem ergeben, dass das Potenzial softwaregestützter Einladungs- und Erinnerungssysteme in Deutschland noch nicht ausreichend ausgeschöpft ist [19].

Befragte Eltern der vorliegenden Stichprobe fanden mehrheitlich, dass es zu wenig öffentliche Aufklärung zur HPV-Impfung gibt. Darüber hinaus fühlte sich nur etwas mehr als die Hälfte der Befragten sehr oder eher gut informiert. Bei Teilgruppen gab es signifikante Unterschiede. Für zukünftige Planungen sollte deshalb die Passgenauigkeit der Maßnahmen hinsichtlich der (Teil‑)Zielgruppen stetig überprüft und ggf. angepasst werden. Zusätzlich zu allgemeinen Informationsangeboten kann auch der Einsatz dialogischer Methoden durch Multiplikatorinnen und Multiplikatoren die Erreichbarkeit von Eltern unterstützen, die sich (noch) nicht gut über die HPV-Impfung informiert fühlen. Das Einbinden der adressierten Zielgruppe und der gemeinsame Austausch können zur Erhöhung der Effektivität von Maßnahmen beitragen [20, 21].

Bei Informations- und Aufklärungsmaßnahmen sollte der Kommunikationsfokus auf dem Schutz vor HPV-bedingten Krebsarten liegen und Mädchen und Jungen bzw. Eltern von Mädchen und Jungen gleichermaßen adressieren.

Ein wesentlicher Grund gegen eine HPV-Impfung ist nach wie vor die Sorge vor möglichen Nebenwirkungen der HPV-Impfung [22, 23]. In der vorliegenden Befragung wurde die Angst vor möglichen Nebenwirkungen als dritthäufigster Grund gegen eine Impfung angegeben – 40 % stimmten diesem Grund voll oder eher zu. Aspekte der Sicherheit erscheinen als zentraler beeinflussender Faktor, wenn es um das Zögern bei der HPV-Impfung geht [24]. Zudem hatte das wahrgenommene Risiko für eine HPV-Infektion im Jugendalter bei der vorliegenden Befragung einen signifikanten Einfluss auf die Impfentscheidung. Auch in weiteren Studien wird von der Bedeutung der Risikoeinschätzung berichtet [22]. Die Weiterentwicklung von effektiven Kommunikationsmaßnahmen zur Förderung einer informierten Impfentscheidung in Bezug auf die HPV-Impfung bleibt demnach weiterhin eine große Herausforderung. Der Aufbau und das Aufrechterhalten von Vertrauen sollten hierbei eine wesentliche Rolle einnehmen [22].

Das größte Vertrauen haben Eltern der vorliegenden Stichprobe in Ärztinnen und Ärzte, besonders in Kinder- und Jugendärztinnen und -ärzte, wenn es um Informationen zur HPV-Impfung geht. Auch andere Studien bestätigen, dass Ärztinnen und Ärzte die zentralen Ansprechpersonen für informierte Impfentscheidungen sind und die Impfbereitschaft maßgeblich beeinflussen [25, 26]. Die aktive Ansprache der HPV-Impfung in der ärztlichen Praxis und deren frühzeitige Durchführung spielen eine wichtige Rolle bei der Erhöhung der noch zu geringen Impfquoten in Deutschland. Informationsmaterialien und passgenaue Fortbildungsangebote können Fachkräfte bei der Impfberatung unterstützen.

Dabei sind effektive Strategien gegen Fehlinformation essenziell, um die Impfbereitschaft zu erhöhen [27]. Die Erhöhung des Wissens und des Bewusstseins über Impfungen hat sich dabei als erfolgreiche Strategie zur Reduzierung von Impfmüdigkeit erwiesen [28]. In der Literatur werden derzeit verschiedene Ansätze für Kommunikationsmaßnahmen in der Impfaufklärung bzw. für die Durchführung des Impfgesprächs in der ärztlichen Praxis diskutiert. Für die Effektivität der einzelnen Ansätze existieren jedoch gemischte Forschungsergebnisse [27].

Vielversprechend wird in der Literatur die Kommunikation mit Fokus auf die vorhandene Evidenz und den wissenschaftlichen Konsens bezüglich Impfungen und Impfmythen dargestellt [27]. Dabei gilt es, aktiv und transparent zu kommunizieren und evidenzbasiert über die Sicherheit und Effektivität sowie mögliche Nebenwirkungen der betreffenden Impfung aufzuklären [29]. Dabei sollten alle Beteiligten in die ärztliche Impfaufklärung eingebunden werden, das heißt sowohl die Eltern als auch die Kinder bzw. Jugendlichen, und eine gemeinsame Entscheidung getroffen werden („shared-decision-making“; [29]).

Wenn Ärztinnen und Ärzte gut informiert und überzeugt von Impfempfehlungen sind, sind sie eher geneigt adäquat dazu aufzuklären [30, 31]. Darüber hinaus kann der Empfehlungsstil der Ärztin oder des Arztes die Impfentscheidung positiv beeinflussen. Beim präsumtiven Empfehlungsstil wird z. B. die Impfentscheidung der informierten Personen bereits angenommen („Es ist Zeit für die HPV-Impfung“) anstatt diese zu erfragen („Möchten Sie Ihr Kind heute gegen HPV impfen lassen?“; [29, 32]).

Ein weiterer Ansatz ist die motivierende Gesprächsführung (Motivational Interviewing). Sie zielt darauf ab, die Entscheidungsfindung zu unterstützen, indem die intrinsische Motivation für Veränderung einer Person gestärkt wird [33]. Dieser Ansatz beinhaltet ein kollaboratives und nondirektives Gespräch, welches geprägt ist von Verständnis und Respekt für das Gegenüber. Die Vermittlung von Wissen wird auf die individuellen Bedürfnisse und Bedenken des Gegenübers angepasst. Mögliche Ambivalenzen sollen im Gespräch exploriert und aufgelöst werden, um die Impfbereitschaft zu erhöhen [33]. Die Evidenz für diese Gesprächstechnik ist vielversprechend und sie wird auch in Schulungsmaterialien der Weltgesundheitsorganisation (WHO) zur Kommunikation von Fachkräften über die HPV-Impfung empfohlen [33, 34].

## Fazit

Die HPV-Impfquoten in Deutschland sind immer noch zu niedrig. Die Gründe dafür sind vielfältig. Neben Bedenken zur Sicherheit bzw. zu Nebenwirkungen der Impfung, spielen u. a. auch der Zeitpunkt der Impfung und der empfundene Mangel an öffentlicher Aufklärung eine wichtige Rolle für die Befragten.

Die Passgenauigkeit von Kommunikationsmaßnahmen zur HPV-Impfung sollte stetig überprüft und ggf. angepasst werden. Der Einsatz dialogischer Methoden durch Multiplikatorinnen und Multiplikatoren kann die Erreichbarkeit von Eltern unterstützen, die sich noch nicht ausreichend zur HPV-Impfung informiert fühlen. Ärztinnen und Ärzte genießen ein hohes Vertrauen und sollten bei der HPV-Impfaufklärung durch Materialien und spezifische Schulungen unterstützt werden. Die HPV-Impfung sollte in der ärztlichen Praxis aktiv angesprochen und möglichst frühzeitig durchgeführt werden. Potenziale von (softwaregestützten) Erinnerungs- und Einladungssystemen sollten ausgeschöpft werden.
